# Current News

**Published:** 1889-04

**Authors:** 


### Current News.

Beware of the Furorè Americana Dentista.

The holders of bridge patents seem to be frightened, as they are resorting to intimidation to help their cause.

Dr. J. N. Crouse has been notified that he is to be made corespondent in a personal suit for damages by the International Tooth Crown Company for *active partisanship* in pushing the interests of the Dental Protective Association.

Dr. Charles D. Cook, Brooklyn, said at a recent meeting of the New York Odontological Society, "when any one tells me I dare not do a given thing, then I begin to seriously think about doing it." You may put my name down on the list of subscribers to the Dental Protective Association.

The indictment of Dr. Henry A. Parr, charged with an assault upon Jackson W. Alward, was dismissed yesterday in the Court of General Sessions by Judge Gildersleeve. The case has attracted considerable attention among the dentists of the city for a year past. Mr. Alward is the agent of the International Tooth Crown Company, and he charged that Dr. Parr was infringing upon the patents belonging to the company. Dr. Parr set up a counter claim, whereupon Mr. Alward called at Dr. Parr's office one day last April and demanded settlement. The testimony given at a trial some months ago showed that Mr. Alward was very noisy, and refused to leave when ordered to do so, and Dr. Parr struck him and then put him out. Alward then brought a suit for $5,000 damages, and also had Dr. Parr indicted. In granting the motion for dismissal Judge Gildersleeve said he thought the assault was excusable.—*N. Y. Daily Herald.*

